# Hypertension is associated with osteoporosis: a case-control study in Chinese postmenopausal women

**DOI:** 10.1186/s12891-021-04124-9

**Published:** 2021-03-07

**Authors:** Hao Chai, Jirong Ge, Li Li, Jianyang Li, Yunjin Ye

**Affiliations:** 1 Fujian University of Traditional Chinese Medicine, Fuzhou, China; 2 Key Research Laboratory of Osteoporosis Syndrome Genomics, Fujian Academy of Chinese Medical Sciences, 282 Wusi Road, Fuzhou, 350003 China

**Keywords:** Osteoporosis, Hypertension, Cardiovascular disease, Bone mineral density, Association

## Abstract

**Background:**

Osteoporosis and cardiovascular disease (CVD) are age-related diseases. It is reported that patients with CVD have a higher risk of bone loss. This retrospective study sought to reveal the association between osteoporosis and CVD in Chinese women. Although epidemiological evidence has indicated a relationship between the two, clinical data in southeast China are lacking.

**Methods:**

In total, 2873 participants completed the baseline survey from January 2007 to October 2019, and 2039 were included in this retrospective study. We divided all subjects into an osteoporosis group and a non-osteoporosis group based on their bone mineral density (BMD). Dual-energy X-ray absorptiometry (DXA) was used to examine BMD. The general information came from the questionnaire survey. Cardiovascular diseases were defined by asking participants at the first visit and checking relevant medical records if they had suffered from hypertension, coronary heart disease, or cerebral infarction.

**Results:**

According to the criterion, the osteoporosis group had 678 subjects, and the non-osteoporosis group had 1361 subjects. Subjects in the osteoporosis group had a significantly higher prevalence of hypertension and coronary heart disease. Besides, the proportion of subjects who drank tea and drank milk were relatively higher in the osteoporosis group. The odds ratio (OR) for suffering from osteoporosis was high if the patients had hypertension.

**Conclusions:**

This study indicated that Chinese postmenopausal women with osteoporosis had a higher prevalence of hypertension. Hypertension was significantly associated with osteoporosis.

## Background

Osteoporosis is a skeletal disease accompanied by low bone mass and a deterioration of the bone microarchitecture, leading to increased bone fragility and risk of fractures [1]. Cardiovascular disease (CVD) is mainly caused by arteriosclerosis, including coronary heart disease, hypertension, cerebral infarction, and other vascular diseases. Osteoporosis and CVD are common clinical diseases. With changing lifestyles and ageing population, the incidence has increased significantly and seriously threatens the physical and mental health of elderly individuals.

It was previously thought that the two were independent of each other, but epidemiological studies have proven a correlation between them [2, 3]. Recently, with further study of the relationship between osteoporosis and CVD, it has been confirmed that they share common risk factors among elderly individuals, especially in postmenopausal women. Age, smoking, lack of physical exercise, vitamin D deficiency, and diabetes mellitus are considered common risk factors for osteoporosis and CVD [4–6]. Some studies have indicated that patients with CVD have a higher risk of bone loss. Besides, individuals with low bone mass have higher mortality from CVD [7]. The possible link between CVD and osteoporosis led us to identify a common pathological basis. The common pathogenesis of vascular calcification and bone mineralization involves oxidative stress, inflammation, and lipid metabolism [8, 9]. In this process, they share common regulatory factors, such as bone morphogenetic proteins (BMPs), osteoprotegerin (OPG), matrix protein Gla (MPG), and tumour necrosis factor-alpha (TNF-α) [10, 11].

Similarly, genetic research has also provided evidence for an association between osteoporosis and CVD. The deletion of specific genes in mice and mutations of critical genes in humans can lead to vascular calcification and early osteoporosis [12]. Research on the relationship between the two is beneficial to prevention and treatment. Specifically, clinical evaluations of CVD patients should consider the measurement of bone mineral density. Additionally, patients with osteoporosis should undergo electrocardiogram examination or Doppler ultrasound, which could have great significance for treating the two diseases [5].

Epidemiology has provided the most direct and considerable evidence for the relationship between osteoporosis and CVD. However, the different methods and populations in reported studies thus far limit the reliability of the results [13]. Few clinical studies have reported from different regions of China. In Tibet, a retrospective study of 99 Chinese Tibetan postmenopausal women with type 2 diabetes indicated that the BMD T-score of the spine and femoral neck was inversely associated with systolic blood pressure (SBP) [14]. Another cross-sectional study of 580 patients from Wuhan, China revealed a correlation between total hip BMD and cardio-ankle vascular index (CAVI) values in middle-aged and elderly inpatients [15]. CAVI is a relatively new non-invasive indicator of arterial stiffness, reflecting the overall arterial elasticity from the origin of the aorta to the ankle artery. Pulse wave velocity (PWV) reflects central and peripheral muscular artery stiffness and is widely used as a marker of arterial stiffness. Brachial-ankle PWV (baPWV) measurement is closely correlated to aortic PWV. Some authors reported that baPWV was elevated in 512 patients with osteoporosis [16]. However, given the relatively small sample size included, it is difficult to reflect the overall situation. These studies also paid more attention to the relationship between disease-related indicators rather than directly reflect the relationship between the two diseases. This retrospective study focuses on postmenopausal women in Fuzhou involving nearly 2,000 participants, which can represent, to a certain extent, the prevalence of osteoporosis in southeastern China. It also provides large sample size data for further investigating the relationship between osteoporosis and CVD in Chinese women.

## Methods

### Study population

This retrospective study was carried out from January 2007 to October 2019 with participants from Fuzhou, capital of Fujian Province. All participants were investigated in the Department of Osteoporosis of the Fujian Academy of Chinese Medical Sciences. The 2873 participants aged between 41 and 90 accepted the questionnaire survey, and 2039 natural postmenopausal women who met the criteria were included in this study. All subjects could provide complete medical records and sign informed consent independently.

All participants were excluded if they had hyperparathyroidism, diabetes, rheumatoid arthritis, or other endocrine and immune diseases that affect bone metabolism. Patients with severe liver, kidney or haematopoietic diseases and malignant tumours were also excluded. Further, none of the subjects had received osteoporosis treatment or other known drugs that affect bone metabolism. No potential subjects had missing key data. Written consent was necessary for all participants.

### Clinical characteristics

The data required for the study came from the questionnaire survey. General information, such as age, height, weight, blood pressure, menarche age, menopausal age, past medical history, and personal lifestyle, was collected and carefully filled in the unified form by trained physicians. Height and weight were measured without shoes, and the body mass index (BMI) was calculated. Before blood pressure measurement, participants needed to rest quietly for at least 5 min, empty their bladders, and not take any drugs. The data used were the average of two measurements.

### Bone mineral density measurement

Dual-energy X-ray absorptiometry (DXA) (Discovery W, Hologic Inc., USA) was used to measure BMD of the lumbar spine and left femoral neck. The T score was calculated according to the reference range of the instrument manufacturer. The inspection was performed daily by the same professional physician. The coefficient of variation (CV) for repeated measurements was approximately 1.0%.

### Definitions

The diagnosis of osteoporosis was based on the T score in the lumbar spine or femoral neck. A T score ≤ − 2.5 indicates osteoporosis, osteopenia is diagnosed as − 2.5 < T score < − 1.0, and a T score ≥ − 1.0 indicates normal [17]. All subjects were divided into the osteoporosis group and non-osteoporosis group according to their T score. Cardiovascular diseases were defined by asking participants at the first visit and checking relevant medical records if they had suffered from hypertension, coronary heart disease, or cerebral infarction, as indicated by International Statistical Classification of Diseases and Related Health Problems (ICD) (tenth revision, ICD-10). Hypertension (ICD-10 code I10) was defined as systolic blood pressure ≥ 140 mmHg and/or diastolic blood pressure ≥ 90 mmHg or intake of antihypertensive drugs. The diagnosis of coronary heart disease (ICD-10 code I25) was based on the guidelines of the European Society of Cardiology and Chinese Society of Cardiology of Chinese Medical Association: the subjects had discomfort related to myocardial ischaemia, including location, character, duration and relationship to exertion and other exacerbating or relieving factors. All subjects had a resting 12-lead ECG recorded allowing for detection of ST-segment changes [18, 19]. The patient was considered to have cerebral infarction (ICD-10 code I63) based on the guidelines of the American Heart Association/American Stroke Association: when no imaging evidence was available, the time limit for the duration of symptoms and signs exceeding 24 h was diagnosed as cerebral infarction [20]. Lifestyle habits were investigated, such as drinking coffee, tea, and milk. Drinkers were defined as women consuming more than 200 ml of tea, coffee, and milk daily and non-drinkers as those consuming less than 200 ml daily or none at all.

### Statistical analysis

EpiDate 2 (the EpiData Association, Odense M, Denmark) was used to enter and proofread data repeatedly, and SPSS 23.0(IBM,Inc., New York, USA) software was used to conduct all analyses. The results are expressed as the average standard deviation or quantity (percentage). We used the independent samples t-test or Mann-Whitney U-test according to whether the continuous variables conformed to the normal distribution. The Pearson chi-square test was used to measure the difference in frequency. Logistic regression analysis was confirmed with osteoporosis as a dependent variable to investigate the factors affecting osteoporosis. First, the independent variables were screened. Univariate regression analysis was used to exclude non-statistically significant variables (*P* > 0.05) and eliminate the mediator variables. Multicollinearity was assessed using stepwise regression. For continuous variables, the linear condition was assessed. The −2 log-likelihood ratio was used to test the overall significance of the model. The Hosmer-Lemeshow test evaluated the model’s goodness-of-fit. All statistical hypothesis tests were two-sided and performed at the 0.05 significance level.

## Results

### Characteristics of subjects in the osteoporosis group and non-osteoporosis group

After excluding 834 participants who did not meet the criteria, 2039 subjects were included in the study. Among these 834 people, 64 were not menopausal, 393 were artificial menopausal, and the rest had other diseases that affect bone metabolism. There were 678 subjects in the osteoporosis group and 1361 subjects in the non-osteoporosis group. The age of the osteoporosis group was between 47 and 90, and the non-osteoporosis group was between 45 and 85. Statistically, the age and menarche age of subjects who suffered from osteoporosis were significantly older than those who did not, and the menopausal age was more advanced. They also had a lower height and lighter weight (*P* < 0.001). In addition, the BMD of the osteoporosis group was significantly lower than the that of the non-osteoporosis group (*P* < 0.001) (Table 1).

**Table 1:** Characteristics of subjects between the osteoporosis group and non-osteoporosis group (mean ± SD)

	Total (*n* = 2039)	Osteoporosis (*n* = 678)	Non-osteoporosis (*n* = 1361)	*P*
Age (years)	62.56 ± 6.67	64.29 ± 6.71	61.70 ± 6.49	< 0.001^a^
Height (m)	1.59 ± 0.05	1.55 ± 0.05	1.56 ± 0.05	< 0.001 ^a^
Weight (kg)	57.43 ± 8.39	56.10 ± 8.10	58.08 ± 8.46	< 0.001 ^a^
BMI (kg/m^2^)	23.63 ± 3.10	23.41 ± 3.06	23.74 ± 3.12	0.023 ^a^
Menarche age (years)	15.39 ± 2.00	15.56 ± 2.10	15.31 ± 1.95	0.021 ^a^
Menopause age (years)	50.33 ± 3.33	50.10 ± 3.40	50.44 ± 3.29	0.036 ^a^
Lumbar spine BMD (g/cm^2^)	0.77 ± 0.15	0.65 ± 0.08	0.83 ± 0.13	< 0.001 ^a^
Femoral neck BMD (g/cm^2^)	0.73 ± 0.14	0.66 ± 0.14	0.77 ± 0.12	< 0.001 ^a^

*Abbreviations*: *BMI* body mass index, *BMD* bone mineral density, *SD* standard deviation

^a^Mann-Whitney U-test

### Comparison of influencing factors between the osteoporosis group and non-osteoporosis group

Comparing the influencing factors between the two groups indicated that patients in the osteoporosis group had a higher prevalence of hypertension and coronary heart disease (*P* < 0.05). Besides, the proportion of subjects who drank tea and drank milk were relatively higher in the osteoporosis group (*P* < 0.05) (Table 2).

**Table 2:** Comparison of influencing factors between the osteoporosis group and non-osteoporosis group (%)

	Total	Osteoporosis	Non-osteoporosis	*P*
N (%)	2039 (100)	678 (33.25)	1361 (66.75)	NA
Drinking coffee, *n* (%)	51 (2.50)	20 (2.95)	31 (2.28)	0.360 ^a^
Drinking tea, *n* (%)	196 (9.61)	79 (11.65)	117 (8.60)	0.027 ^a^
Drinking milk, *n* (%)	633 (31.04)	276 (40.71)	357 (26.23)	< 0.001 ^a^
Hypertension, *n* (%)	359 (17.61)	151 (22.27)	208 (15.28)	< 0.001 ^a^
Coronary heart disease, *n* (%)	104 (5.10)	50 (7.37)	54 (3.97)	0.001 ^a^
Cerebral infarction, *n* (%)	22 (1.08)	9 (1.33)	13 (0.96)	0.443 ^a^

*Abbreviations*: *NA* not applicable

^a^ Pearson Chi-square test

### Logistic regression analysis for the effect of independent variables

Univariate regression analysis was used to exclude non-statistically significant variables, including coffee consumption and cerebral infarction (*P* > 0.05). The variables selected for the regression model were age, height, weight, BMI, menarche age, menopausal age, drinking tea, drinking milk, hypertension, and coronary heart disease. Multivariate regression analysis showed that hypertension was a significant influencing factor for osteoporosis (*P* < 0.05). In addition, increased age, menarche age, and milk consumption were related to an increased risk of osteoporosis. Moreover, lower height, lighter weight, and earlier menopause may also be related to the increased risk of osteoporosis (Table 3).

**Table 3:** Multivariate Logistic regression analysis for the effect of independent variables

	Univariate Logistic regression			Multivariate Logistic regression		
	OR	95% CI	*P*	OR	95% CI	*P*
Age (year)	1.061	1.046–1.076	< 0.001	1.047	1.031–1.063	< 0.001
Height (m)	0.003	0.001–0.020	< 0.001	0.053	0.007–0.421	0.006
Weight (kg)	0.971	0.960–0.982	< 0.001	0.979	0.967–0.992	0.002
BMI (kg/m^2^)	0.966	0.937–0.996	0.024			
Menarche age (year)	1.063	1.015–1.113	0.009	1.061	1.012–1.114	0.015
Menopause age (year)	0.970	0.944–0.998	0.033	0.960	0.933–0.988	0.006
Drinking coffee	1.303	0.737–2.304	0.363			
Drinking tea	1.401	1.036–1.894	0.028			
Drinking milk	1.588	1.592–2.353	< 0.001	1.832	1.495–2.246	< 0.001
Hypertension	1.558	1.258–2.006	< 0.001	1.303	1.012–1.677	0.040
Coronary heart disease	1.927	1.297–2.864	0.001			
Cerebral infarction	1.395	0.593–3.280	0.445			

*Abbreviations*: *BMI* body mass index, *OR* odds ratio, *CI* confidence interval

## Discussion

With the prolongation of life expectancy, the incidence of CVD and osteoporosis is increasing. More evidence has shown common risk factors and similar pathological mechanisms between the two diseases [21]. In this retrospective study, we found that there were 678 cases of osteoporosis among all the women who met the criteria, with a prevalence rate of 33.25%, which was similar to that reported in other studies [22, 23]. Among the 678 patients, 151 suffered from hypertension, and 50 suffered from coronary heart disease. The prevalence was significantly higher than that of non-osteoporosis. This illustrates that CVD may increase the risk of osteoporosis, which is consistent with previous studies [24–26]. To further investigate the factors causing this increased risk, we conducted a multivariate regression analysis and found that hypertension was related to an increased risk of osteoporosis. This indicated a significant association between hypertension and osteoporosis, suggesting that fracture and CVD prevention should be considered when treating osteoporosis. According to the results, drinking milk also increased the risk of osteoporosis, which was inconsistent with previous studies. This may be related to our failure to record the duration and quantity of milk.

Some studies have reported that low BMD is superior to traditional factors, such as hyperlipidaemia and smoking, in predicting the development of cardiovascular events [27, 28]. The lower BMD and increased bone loss rates were also associated with an increased risk of CVD in the Chinese cohort [29]. Interestingly, a study showed a favourable relationship between a reduced risk of cardiovascular disease and BMD [30]. Another study followed 6872 men and women for 5.7 years, and 196 developed myocardial infarction during this period. The results revealed that low hip BMD was an influencing factor for infarction [31]. These studies showed the relationship between low BMD and CVD. Low BMD is a significant phenotype of osteoporosis, illustrating the relationship between osteoporosis and CVD.

Osteoporosis and CVD are common age-related diseases. As shown in the results, the risk of osteoporosis increased with age. Changes in oestrogen levels caused by menopause or ageing can directly affect blood vessel walls and bone metabolism [15]. Bones and blood vessels are considered to be important targets for oestrogen, which can improve the function of endothelial cells and vascular smooth muscle cells, inhibit platelet aggregation, and affect blood vessel responses to injury [32, 33]. Similarly, oestrogen in serum can reduce the number and activity of osteoclasts and inhibit bone resorption. The decrease in oestrogen results in increased bone resorption [34–36]. In addition, the reduction may lead to an increase in proinflammatory cytokines, which are related to bone loss and severe arteriosclerosis [37, 38].

Disorders of lipid metabolism in postmenopausal women are closely related to bone dysfunction [39]. Dyslipidaemia is also one of the pathogenic factors of hypertension, which may explain the relationship between osteoporosis and hypertension. Our results also showed that hypertension is a significant influencing factor for osteoporosis. Many clinical studies support the effect of lipid metabolism on osteoporosis [40]. A survey of Chinese people showed that elevated levels of serum high-density lipoprotein cholesterol (HDL-C) led to higher risk of osteoporosis, but a higher HDL-C level was favourable for cardiovascular diseases [41]. Another study indicated that femoral neck BMD in postmenopausal women was positively correlated with low-density lipoprotein cholesterol (LDL-C) and negatively correlated with HDL-C [42]. Some clinical data from other populations also support this view [43–45]. An animal study demonstrated that an atherogenic diet could lead to lower bone mineral content (BMC) and BMD [46]. Some cytokines secreted by adipose tissue have also been shown to be involved in the regulation of bone metabolism, such as leptin and adiponectin [40]. All these studies illustrate the correlation between lipid and bone metabolism.

A limitation of this study is that it was a single-centre retrospective study with poor homogeneity, many confounding factors, and low evidence. Second, it was difficult to obtain a causal relationship between influencing factors and osteoporosis; thus, a prospective cohort study should be considered in the future. Third, the information related to cardiovascular disease came from medical records rather than immediate examination. Although we tried our best to obtain objective data, there were still some biases in personal lifestyle data. Further research needs to be performed based on this study.

## Conclusion

In short, this observational study indicated an association between osteoporosis and CVD in Chinese postmenopausal women. Based on the results, cardiovascular assessment should be considered in patients with osteoporosis to prevent adverse events. This further supports the view that there is a biological link between the two.

## Data Availability

The datasets generated and/or analysed during the current study are not publicly available due to personal protection laws. Subsets or aggregation of these data will not include information that could compromise research participants’ privacy and are available from the corresponding author on reasonable request.

## Abbreviations

CVD: Cardiovascular disease
BMP: Bone morphogenetic proteins
OPG: Osteoprotegerin
MPG: Matrix protein Gla
TNF-a: Tumor necrosis factor-alpha
BMD: Bone mineral density
DXA: Dual-energy X-ray absorptiometry
CV: Coefficient of variation
NA: Not applicable
BMI: Body mass index
SD: Standard deviation
SE: Standard error
OR: Odds ratio
CI: Confidence interval
HDL-C: High-density lipoprotein cholesterol
LDL-C: Low-density lipoprotein cholesterol
BMC: Bone mineral content
